# Occult femoral neck fractures in ipsilateral femoral shaft and patellar fractures: A case series about diagnostic challenges and proposed strategies

**DOI:** 10.1016/j.tcr.2025.101260

**Published:** 2025-11-13

**Authors:** Yu-Chao Lin, Wei-Long Lin, Jun-Lin Liu, Yu-Dong Huang, Xiao-Hua Zheng

**Affiliations:** a The 95th Hospital of Putian, No. 485, Dongyan Road, Licheng District, Putian City, Fujian Province, 351100, China^1^1 Present address.; b Sichuan University West China Hospital Tibet People's Government in Chengdu Office Branch, No. 20, Ximianqiao Hengjie, Wuhou District, Chengdu City, Sichuan Province, 610041, China

**Keywords:** Ipsilateral, Femoral shaft fracture, Patellar fracture, Occult femoral neck fracture, Missed diagnosis, Revision surgery, Intramedullary nailing

## Abstract

This case series presents two patients with high-energy trauma resulting in ipsilateral femoral shaft and patellar fractures. Preoperative CT imaging suggested a bone cyst but failed to detect an associated femoral neck fracture, which was also missed during intraoperative fluoroscopy and identified only on postoperative review. Both patients underwent revision surgery involving repositioning of the proximal intramedullary nail locking screws to achieve fixation directed into the femoral neck. At the 6-month follow-up, fracture healing was satisfactory with no signs of avascular necrosis of the femoral head. This report highlights the risk of missed occult femoral neck fractures in high-energy trauma and proposes a diagnostic workflow including MRI screening for femoral neck injury in cases of high-energy lower limb trauma. The efficacy of revision nailing with recon locking is also demonstrated.

## Introduction

The incidence of ipsilateral femoral neck fractures with femoral shaft fractures ranges from 1 % to 9 %. Occult femoral neck fractures are particularly susceptible to underdiagnosis, with missed rates exceeding 30 % due to the limited sensitivity of radiography and CT [1]. Occult femoral neck fractures represent an even smaller subset of these cases, and they are less frequently identified and intervened upon at an early stage [43,44].Failure to achieve timely fixation may lead to nonunion, avascular necrosis of the femoral head, and hip dysfunction. This article analyzes two cases of missed occult femoral neck fractures in patients with ipsilateral femoral shaft and patellar fractures, with discussion of injury mechanisms, diagnostic strategies, and revision techniques to raise clinical awareness.

## Case presentation

### Case 1

A 32-year-old male was injured in a high-speed motorcycle collision, striking a guardrail primarily with his left knee, with the hip flexed and abducted.

Physical examination revealed deformity and swelling of the left mid-thigh, patellar skin contusion without active bleeding, knee swelling, positive axial tenderness, negative hip compression and percussion tests, restricted knee motion, and normal pedal pulses.

### Case 2

A 45-year-old male fell from a rooftop while working in a squatting position, landing directly on his right knee with the hip flexed and abducted.

Examination showed deformity and swelling of the right mid-thigh, patellar contusion, knee swelling, positive axial tenderness, negative hip stress tests, limited knee motion, and normal neurovascular status.

**Fig. 7:**
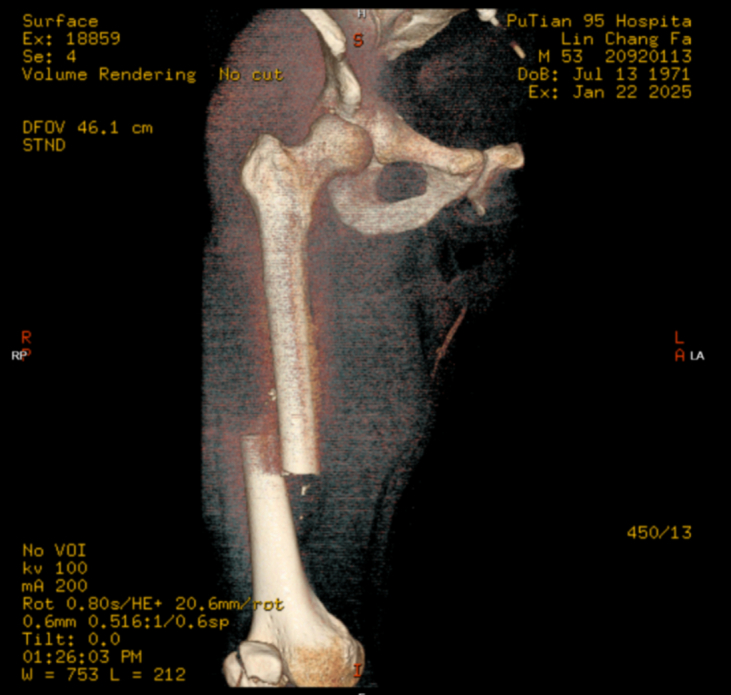
Preoperative CT identified a distal femoral shaft fracture (OTA/AO 32A) and a patellar fracture (OTA/AO 34-C3).

**Fig. 8:**
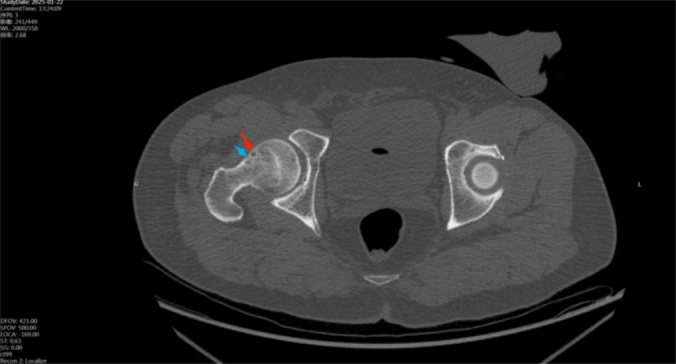
Preoperative hip CT revealed no definite fracture line in the femoral neck. A small cystic lucency (∼3 mm) was noted in the anterior midportion of the femoral neck, suggestive of a bone cyst or synovial hernia pit (arrow).

**Fig. 9:**
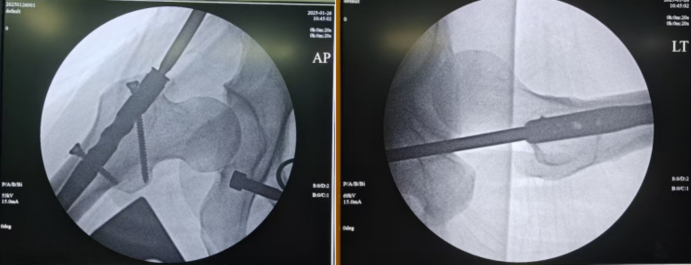
Intraoperative fluoroscopy was negative.

**Fig. 10:**
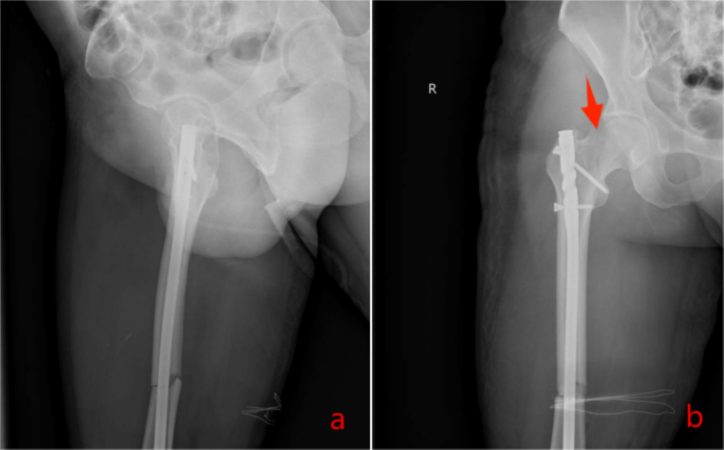
Postoperative day 2 X-ray showed a basicervical femoral neck fracture (Pauwels type III).

**Fig. 11:**
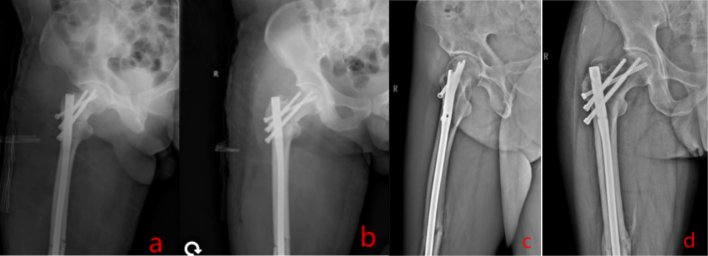
Revision fixation was performed (a & b). And the reexamination 6 months after the operation (c & d).

**Fig. 12:**
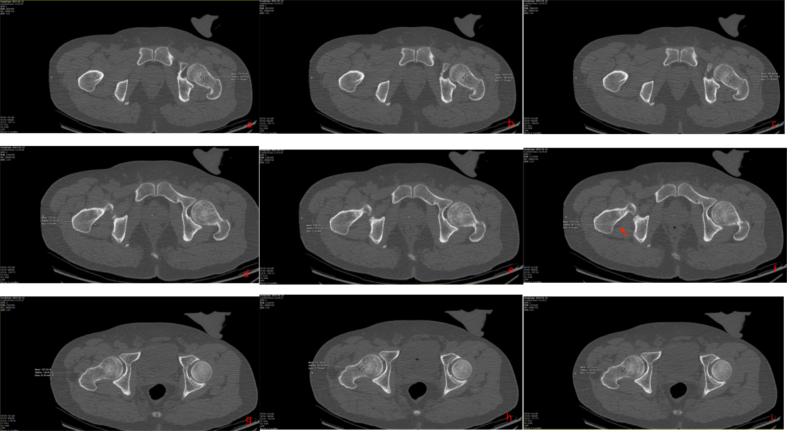
Comparative CT attenuation measurements are presented.

**Table 1:** Measurement results of bone density CT values (Unit: HU).

Case	Side type	ROI (mm^2^)	CT value			Mean ± SD
			Result 1	Result 2	Result 3	
Case 1	Healthy side	52.6	140.22	142.94	144.09	142.42 ± 1.95
	Affected side		97.90	88.04	109.33	98.42 ± 10.65
Case 2	Healthy side	53.1	254.38	289.67	264.08	269.38 ± 18.75
	Affected side		187.25	171.38	187.56	182.06 ± 9.21

Case 1: affected vs. unaffected side, *t* = 7.4897, *p* = 0.0174; Case 2: *t* = 5.5530, *p* = 0.0309. Differences were statistically significant (p < 0.05). Data are expressed as mean ± SD.

**Table 2:** Measurement results of CT values of soft tissues in the affected joint capsule (Unit: HU).

Case	ROI (mm^2^)	CT value			Mean ± SD
Case 1	8.0	46.64	45.00	–	45.82 ± 1.16
Case 2	8.0	3.82	9.09	10.91	7.94 ± 3.74

Bone density measurements avoided Ward's.

## Discussion

With the increasing number of traffic accidents, high-energy injuries are becoming more common. In polytrauma patients, severe pain from obvious injuries often masks occult fractures, leading to delayed diagnosis and treatment, which may result in serious complications [2]. Literature suggests that when the lower limb is in a flexed knee and hip position, high-energy force applied to the anterior knee can cause patellar and femoral shaft fractures. Most energy is absorbed at the femoral shaft level, and the residual force transmitted upward may cause hip injury. When the hip is flexed and abducted, the femoral head is relatively stable within the acetabulum, making dislocation less likely but predisposing to femoral neck fracture [3,4], consistent with our two cases. Both patients sustained axial load on the flexed knee, resulting in patellar and femoral comminuted fractures that dissipated most of the energy. The remaining axial and abduction forces transmitted upward caused compressive and rotational stress on the femoral neck, leading to incomplete fracture or bone marrow edema.

In high-energy trauma, occult injuries are easily overlooked. Among patients with ipsilateral femoral shaft and knee injuries, 20 %–50 % have delayed diagnosis of an ipsilateral femoral neck fracture [1,[5], [6], [7]]. In our cases, the patients reported severe thigh and knee pain but did not initially describe hip pain. During physical examination, percussion of the greater trochanter elicited significant thigh pain, distracting from hip discomfort. Initial imaging often consists of X-rays or CT [[8], [9], [10]], yet the sensitivity of these modalities for occult femoral neck fractures is limited (56 %–64 %) [11,12]. In our cases, preoperative hip CT included 1 mm coronal, sagittal, axial, and 3D reconstructions. Multiple physicians reviewed the images but identified no continuous fracture line—only a small bone cyst. Retrospective review with adjusted window settings revealed a fracture line traversing the cyst (Fig. 2, Fig. 8).Studies indicate that bone cysts may obscure microfractures and weaken proximal femoral stability under torsional stress, predisposing to occult fractures [[13], [14], [15]].

We did not routinely measure CT Hounsfield units (HU) in the femoral neck preoperatively. CT HU values can effectively assess bone density and osteoporosis risk. A value below 147.4 ± 35.5 HU in a 5 mm ROI may indicate osteopenia or localized bone loss [[16], [17], [18], [19], [20], [21]], warranting further MRI evaluation. MRI—particularly coronal T1-weighted and STIR sequences—is 100 % sensitive for detecting radiographically occult femoral neck fractures [[22], [23], [24], [25], [26]]. Soft tissue findings were also overlooked; prior studies suggest that the “capsular sign” (fat-fluid level) on CT in high-energy femoral shaft fractures may indicate occult ipsilateral femoral neck fracture, supporting preoperative hip MRI to avoid unplanned surgery [38,39].

In retrospective analysis, we measured CT values in both affected and unaffected femoral necks using a 64-detector scanner (GE Lightspeed VCT-64) at 120 kV with automatic tube current and 1 mm slice thickness. On coronal images aligned with the femoral neck axis, elliptical ROIs >50 mm^2^ were placed within the trabecular bone >2 mm from the cortex. Mean HU values were obtained from three consecutive slices (Fig. 6, Fig. 12). In Case 1, the affected femoral neck measured 98.42 ± 10.65 HU versus 142.42 ± 1.95 HU on the contralateral side. A cystic area near the anterior capsule measured 45.82 ± 1.16 HU, consistent with fluid. In Case 2, the affected side measured 182.06 ± 9.21 HU compared to 269.38 ± 18.75 HU on the healthy side. A posterior capsular cyst measured 7.94 ± 3.74 HU. Differences between sides were statistically significant (*p* < 0.05) (Refer to Table 1 and Table 2 for detailed data.). We recommend routine measurement of femoral neck and periarticular soft tissue HU values in high-energy trauma patients with ambiguous symptoms. Significantly reduced HU values may indicate a need for MRI to exclude occult fracture.

**Fig. 1:**
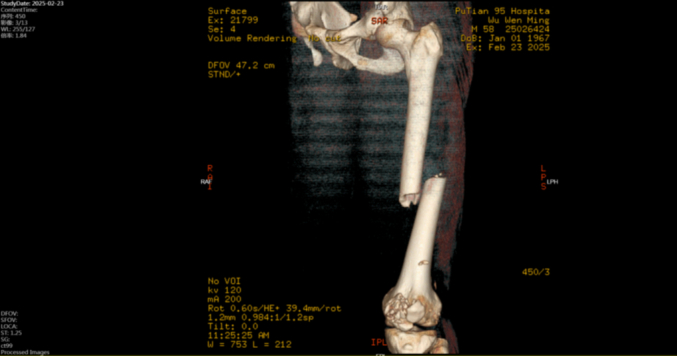
Preoperative CT showed a comminuted mid-shaft femoral fracture (OTA/AO 32A) and a comminuted patellar fracture (OTA/AO 34-C3).

OTA/AO classification of the femoral shaft fracture may help predict associated femoral neck injury. Type 32A fractures are more frequently associated with ipsilateral femoral neck fractures (IFNF) than type 32B [27,28]. Both present cases were classified as OTA/AO 32A (Fig. 1, Fig. 7), underscoring the need for high suspicion of occult femoral neck fracture in such patterns.

**Fig. 2:**
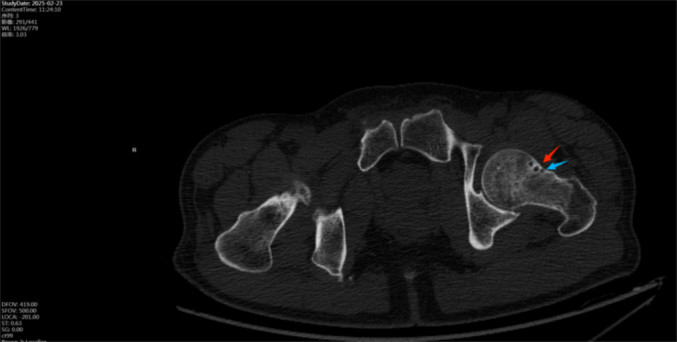
Preoperative hip CT revealed no definite fracture line in the femoral neck. A small cystic lucency (∼3 mm) was noted in the anterior midportion of the femoral neck, suggestive of a bone cyst or synovial hernia pit (arrow).

**Fig. 3:**
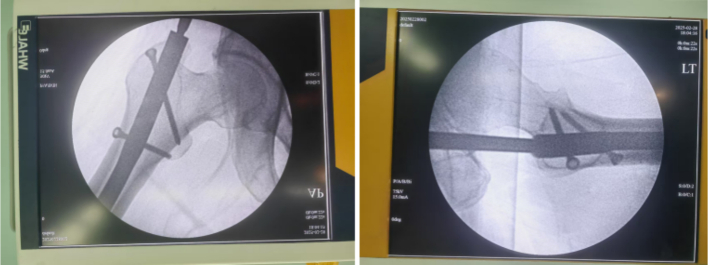
No fracture was visible on intraoperative fluoroscopy.

**Fig. 4:**
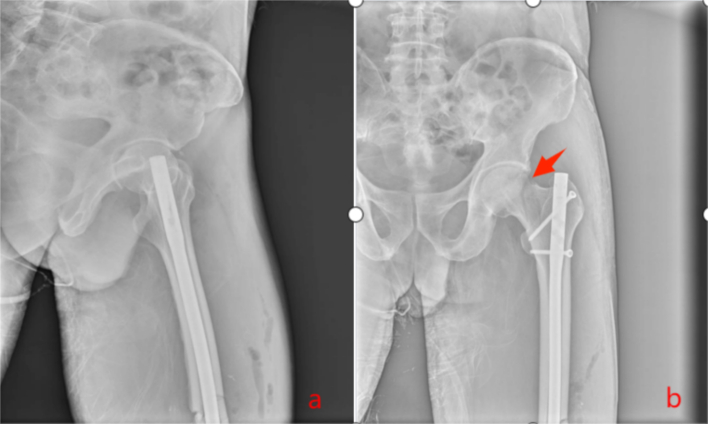
Postoperative day 2 radiographs revealed an incomplete subcapital femoral neck fracture (Pauwels type III).

**Fig. 5:**
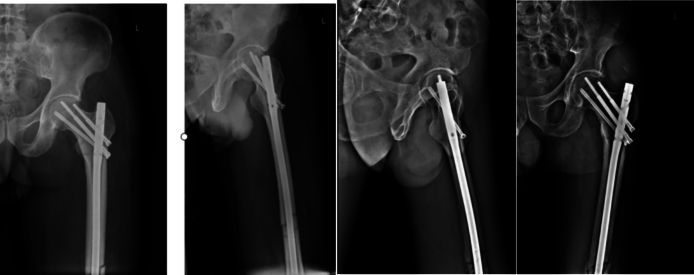
Revision involved recon locking of the intramedullary nail.And the reexamination 2 months after the operation.

**Fig. 6:**
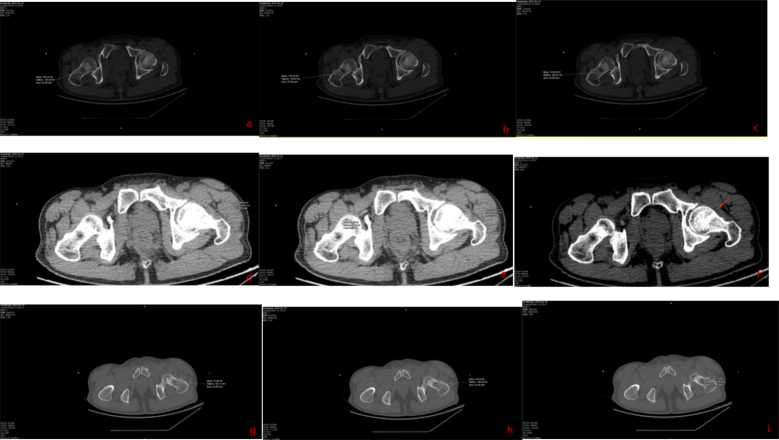
Retrospective CT attenuation measurements were performed around the hip joint.

Untreated occult femoral neck fractures may lead to displacement, nonunion, and avascular necrosis [29]. Early surgical intervention reduces the risk of nonunion and other complications compared to delayed treatment [30,31]. Prompt recognition and fixation are essential for functional recovery [32,33]. In our cases, initial surgery involved closed reduction and intramedullary nailing (static locking) of the femoral shaft fracture, plus tension band wiring of the patella. Intraoperative fluoroscopy did not reveal the femoral neck fracture (Fig. 3, Fig. 9). Postoperatively, patients underwent rehabilitation including active and passive exercises such as straight leg raise and hip/knee flexion. Patients reported hip pain exacerbated by movement. We suspect that the occult femoral neck fractures progressed to incomplete separation during rehabilitation.Repeat imaging within one week confirmed the diagnosis (Fig. 4, Fig. 10). Revision surgery was performed promptly, and at 6-month follow-up, both fractures had healed without signs of avascular necrosis, with excellent Harris Hip Scores (Fig. 5, Fig. 11). However, given that the peak incidence of traumatic avascular necrosis (AVN) of the femoral head typically occurs within two years postoperatively, close monitoring during this period is essential for early detection of AVN progression [40,41]. Furthermore, assessing the risk of AVN requires long-term observation, with a critical window extending up to five years. Therefore, we strongly recommend continued follow-up for at least five years or longer in such cases [42].

There is no consensus on the optimal revision technique [34]. We replaced the static locking screw with two recon locking screws directed into the femoral neck and added a cannulated compression screw alongside the nail to improve rotational stability and reduce shear stress while preserving axial load-sharing [37]. The original surgical approach was reused to minimize additional trauma. Preoperative discussion with patients regarding the risk of occult fracture can facilitate informed consent and improve compliance with subsequent revision [35,36].

Based on these experiences, we propose the following optimized diagnostic pathway:

**Figure:**
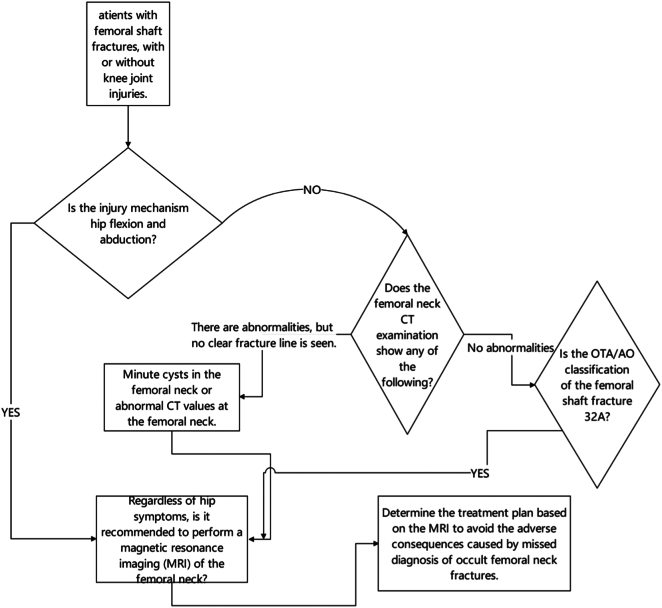

## Conclusion

In high-energy femoral shaft injuries, clinicians should maintain a high index of suspicion for ipsilateral occult injuries—especially with a mechanism involving hip flexion and abduction, concomitant knee injury, and OTA/AO type 32A fracture pattern. The possibility of occult femoral neck fracture should be thoroughly communicated with patients. CT alone is insufficient to exclude occult fracture. Although not routine, preoperative hip MRI is a reliable screening tool. Cystic changes or significantly reduced CT HU values in the femoral neck or periarticular soft tissue should prompt MRI evaluation to avoid missed diagnosis and enable timely intervention. If a femoral neck fracture is detected postoperatively, early revision with recon locking screws can achieve stable fixation, minimize additional trauma, and prevent catastrophic complications. Both patients presented had satisfactory outcomes at 6 months, although longer follow-up (>2 years) is necessary to monitor for delayed avascular necrosis. Whether CT HU values can reliably predict occult fractures requires validation in larger studies.

## CRediT authorship contribution statement

**Yu-Chao Lin:** Writing – review & editing, Writing – original draft, Methodology, Data curation, Conceptualization. **Wei-Long Lin:** Writing – review & editing, Data curation, Conceptualization. **Jun-Lin Liu:** Writing – review & editing, Data curation, Conceptualization. **Yu-Dong Huang:** Investigation, Data curation. **Xiao-Hua Zheng:** Data curation.

## Consent to participate

Patient consented for participation.

## Consent for publication

The patient consented for publication.

## Clinical trial number

Not applicable.

## Ethics approval

Approval from ethics committee was not required.

## Funding

No funding was inflicted.

## Declaration of competing interest

The authors declare that they have no competing interests.

## Data Availability

All data generated or analysed during this study are included in this published article.
